# Nasopharyngeal Microbiota in SARS-CoV-2 Positive and Negative Patients

**DOI:** 10.1186/s12575-021-00148-6

**Published:** 2021-06-01

**Authors:** Phillip A. Engen, Ankur Naqib, Cheryl Jennings, Stefan J. Green, Alan Landay, Ali Keshavarzian, Robin M. Voigt

**Affiliations:** 1 Rush Medical College, Rush Center for Integrated Microbiome and Chronobiology Research, Rush University Medical Center, 1725 W. Harrison St. STE 206, Chicago, IL 60612 USA; 2 Department of Molecular Pathogens and Immunity, Rush University Medical Center, Chicago, IL USA; 3 Genomics and Microbiome Core Facility, Rush University Medical Center, Chicago, IL USA; 4 Department of Internal Medicine, Division of Geriatrics and Palliative Medicine, Rush Medical College, Chicago, IL USA; 5 Department of Physiology, Rush University Medical Center, Chicago, IL USA; 6 Department of Medicine, Rush University Medical Center, IL Chicago, USA

**Keywords:** SARS-CoV-2, COVID-19, Nasopharyngeal, Microbiota, Dysbiosis

## Abstract

We investigated nasopharyngeal microbial community structure in COVID-19-positive and -negative patients. High-throughput 16S ribosomal RNA gene amplicon sequencing revealed significant microbial community structure differences between COVID-19-positive and -negative patients. This proof-of-concept study demonstrates that: (1) nasopharyngeal microbiome communities can be assessed using collection samples already collected for SARS-CoV-2 testing (viral transport media) and (2) SARS-CoV-2 infection is associated with altered dysbiotic microbial profiles which could be a biomarker for disease progression and prognosis in SARS-CoV-2.

**Supplementary Information:**

The online version contains supplementary material available at 10.1186/s12575-021-00148-6.

## Introduction

Since the appearance of severe acute respiratory syndrome coronavirus 2 (SARS-CoV-2) infections in 2019, cases of coronavirus disease 2019 (COVID-19) have spread rapidly around the world infecting over 152 million people and claiming over 3.2 million lives, as of May 3, 2021 (https://covid19.who.int/). COVID-19 is primarily transmitted through the respiratory tract via aerosolized droplets containing viral particles [1]. Host-associated microorganisms can influence viral infectivity [2] and are the major players mediating immune-inflammation [3]. Conversely, studies have shown that viruses can modulate microbiota community in the oropharyngeal and respiratory tract [4]. However, few studies have examined the nasopharyngeal microbiota in COVID-19 patients. To date, two studies found no significant differences in microbial diversity between COVID-19-positive and negative patients [5, 6] whereas two studies did note differences in community structure [7, 8]. One of these studies reports a significant decrease in microbial diversity, differences between microbial communities, and a higher abundance of *Propionibacteriaceae* and a reduction in *Corynebacterium accolens* in COVID-19-positive relative to -negative patients [7]. The other study observed differences between microbial communities, with a significantly lower abundance of *Fusobacterium periodonticum* in COVID-19 positive compared to negative patients [8]. These mixed results highlight the critical need for more information.

This proof-of-concept study utilized viral transport media (VTM) used for SARS-CoV-2 nasopharyngeal swab sample collection/testing to assess nasopharyngeal microbiota communities in COVID-19-positive and negative patients. Given the critical importance of understanding the nasopharyngeal microbiome in SARS-CoV-2 infection and COVID-19, use of alternate sources, like VTM, is essential.

## Methods

### Sample Cohort, Collection, Extraction and Sequencing

We examined COVID-19-positive (*n* = 9) and -negative (*n* = 10) subjects recruited from Rush University Medical Center (RUMC) located in Chicago, IL in the early stage of the pandemic (April 2020). Based on the Centers for Disease Control and Prevention (CDC) classification, the nine COVID-19-positive subjects had mild COVID-19 with no hospitalizations or deaths reported (See Supplementary Table 1, Additional File 1). The study was approved by the RUMC Institutional Review Board [COVID-19 Biorepository (ORA #20,032,309)] to use random de-identified remnant specimens from standard care testing of COVID-19. There were no a priori exclusion criteria.

The feasibility of using nasopharyngeal swab (NPS) VTM collection for SARS-CoV-2 detection and microbiome analysis has been previously reported [9]. The NPS were collected according to CDC guidelines. Sterile synthetic-head, plastic-shaft swabs were used to collect specimens for diagnostic testing and were placed into properly labeled collection tubes containing 3 mL of VTM [REMEL Micro Test™ M4RT®]. The NPS were sent to the RUMC clinical microbiology laboratory and heat inactivated [65˚C for (30) minutes] prior to RT-PCR testing on an Abbott *m*2000 device [Abbott Laboratories] [10]. Due to the surge in testing and specimen processing backlog in the early stage of the pandemic the NPS tubes were stored at 4˚C for up to nine days prior to processing for storage. The NPS + VTM tubes were vortexed briefly for (3) seconds and the VTM was aliquoted into multiple 250 µl aliquots, and stored at -80˚C.

Isolation of viral nucleic acids and bacterial DNA from nasal swab VTM (200ul) samples were performed using the NucleoMag Pathogen manufacturer’s protocol (Macherey–Nagel, Duran, Germany). Microbiome characterization was performed using a PCR-next-generation sequencing (NGS) approach with a two-stage PCR protocol, as described previously [11]. The V4 variable region of microbial 16S rRNA genes was amplified with the 515F/806R primer set (515F:GTGYCAGCMGCCGCGGTAA; 806R:GGACTACNVGGGTWTCTAAT) [12] and using Fluidigm Access Array primers for Illumina sequencers. Negative controls (*i.e.*, PCR reagent blanks; *n* = 5) were amplified and sequenced with samples. Sequencing was performed using an Illumina MiniSeq with a mid-output kit and paired-end 153 base reads.

### Analysis of Microbial 16S rRNA Gene Amplicon Sequences

Raw sequences obtained from the sequencer were merged using the PEAR (Paired-End read merger) algorithm (v0.9.11) [13]. Merged sequences were then quality filtered and denoised using the DADA2 algorithm within the QIIME2 (v 2020.8.0) workflow [14, 15]. Amplicon sequence variants (ASVs) were generated and utilized for all downstream analyses. Taxonomy was assigned to ASVs by using the naïve Bayes taxonomy classifier trained with the SILVA 138 99% OTU database [16, 17]. A total of 527,628 sequencing clusters were generated, with an average depth of 27,770 sequences per sample (median = 15,318; min = 5,164; max = 145,270). Four reagent contaminant ASVs were identified and removed using decontam package based on the prevalence of the ASVs in the reagent negative blank controls, using default parameters [18]. Unassigned ASVs and chloroplast and mitochondrial ASVs were removed from statistical analyses [19].

### Statistical Analysis

Analyses of alpha- and beta-diversity were used to compare nasopharyngeal microbial communities in COVID-19-positive and -negative patients; all analyses were performed on feature (ASV) counts. Alpha-diversity metrics were calculated on rarefied data (5,000 sequences/sample). Differences in alpha-diversity indices and bacterial ratios were assessed for significance using unpaired t-test or Mann–Whitney test, based on the outcome of Shapiro-Wilks normality test. Significance levels were set at *p* < 0.05. Permutation Multivariate Analysis of Variance (PERMANOVA) was used to assess global differences in microbial communities between groups [20]. Significance of PERMANOVA values were determined using 9,999 permutations and adjustment for multiple testing was conducted using the Benjamini–Hochberg false-discovery rate correction. Visualization of data was performed using principal coordinates analysis (PCoA) based on a Bray–Curtis dissimilarity distance matrix within the software package QIIME2.

Random forest models (number of runs = 1,023) were used to predict featured taxa of importance using the R version of the Boruta algorithm [21]. Analysis of composition of microbiomes (ANCOM) was performed on microbial communities to identify differentially abundant taxa between groups [22]. Additionally, differential abundances of individual taxa between groups were determined using differential abundance analysis (DESeq2) generating a q-value [23], as DESeq2 has increased sensitivity on smaller datasets (< 20 samples).

## Results

**Fig. 1:**
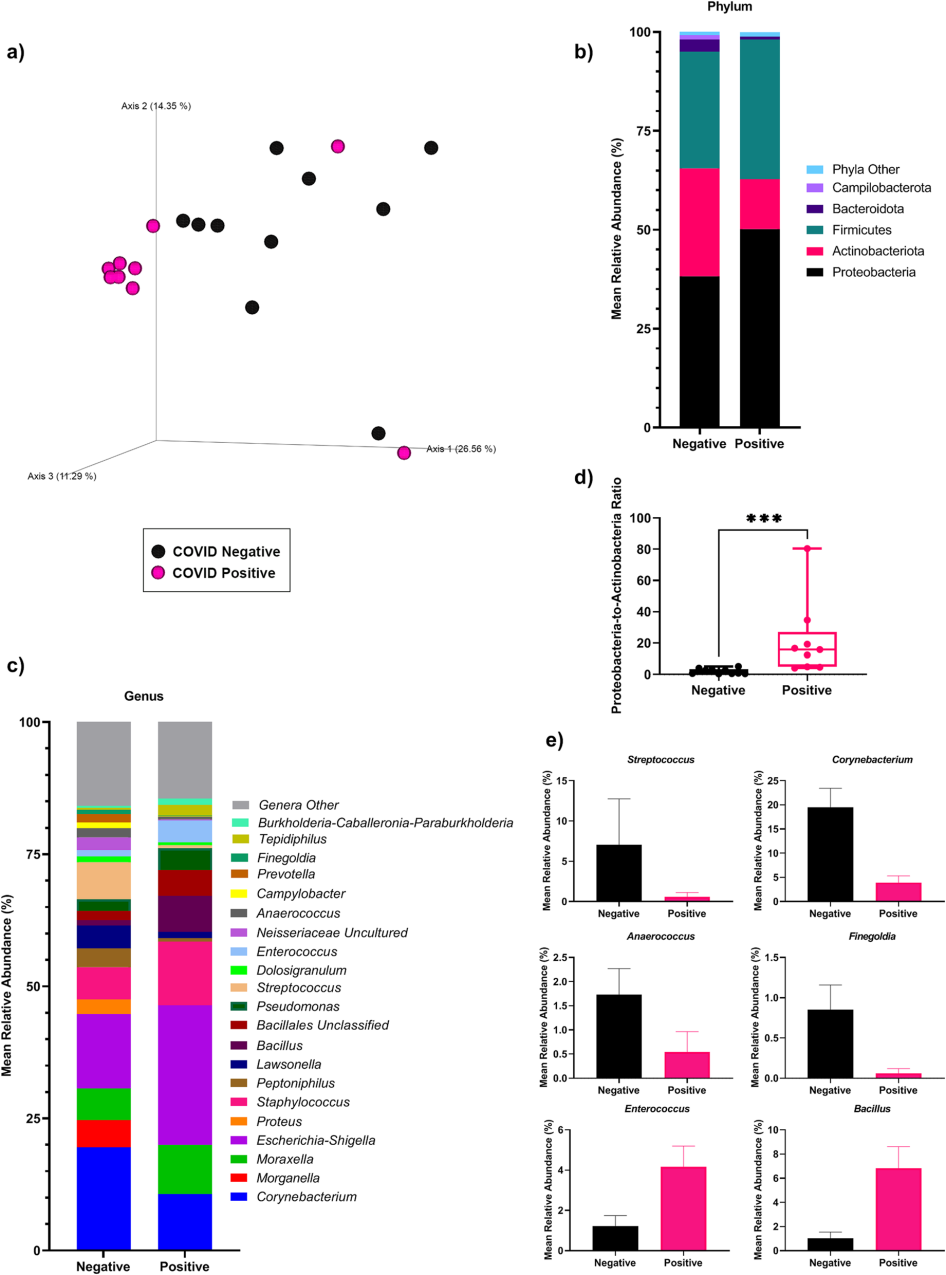
Microbial profiles of the COVID-19-positive and –negative patients. **A** Principal coordinate analysis (PCoA) of nasopharyngeal microbial communities in patients with and without SARS-CoV-2. Communities were significantly different between groups, as assessed by PERMANOVA (*q* = 0.016). **B** Stacked column plots depicting the average relative abundance of bacterial phyla in COVID-19-positive and -negative patients. **C** Stacked column plots depicting the average relative abundance of bacterial genera in COVID-19-positive and -negative patients. **D** Ratio of Proteobacteria-to-Actinobacteria in COVID-19-positive and -negative patients (Mann–Whitney test; *p* = 0.0002). **E** Relative abundance and standard deviation of nasopharyngeal genera identified in ANCOM analyses in COVID-19-positive and -negative patients

Analysis of communities using 16S ribosomal RNA gene amplicon sequencing revealed that microbial alpha-diversity indices were not significantly different between patient groups, but taxonomic feature richness was lower in COVID-19-positive compared to -negative patients (See Supplementary Table 2, Additional File 1). Significant differences (PERMANOVA: *q* = 0.016) in nasopharyngeal microbial community structure were observed between positive and negative patients in beta diversity analyses conducted on microbial features (i.e., amplicon sequence variants, ASVs) (Fig. 1A; See Supplementary Table 3, Additional File 1). Microbial communities in both groups were dominated by the phyla Proteobacteria, Actinobacteria and Firmicutes, and the genera *Corynebacterium*, *Morganella*, *Moraxella*, *Escherichia-Shigella*, *Proteus*, and *Staphylococcus* (˃ 50% of all sequences; Fig. 1B-C; See Supplementary Table 4, Additional File 1). The microbial community in COVID-19-positive patients can be characterized as pro-inflammatory as exemplified by significantly higher (Mann–Whitney test: *p* = 0.0002) Proteobacteria-to-Actinobacteria ratio relative to COVID-19-negative patients (Fig. 1D). Between-group differences in taxon abundances were identified using ANCOM (Table 1) and DESeq2 (See Supplementary Tables 5–6, Additional File 1). ANCOM identified the genus *Streptococcus* as significantly (*q* ˂ 0.05) less abundant in COVID-19-positive relative to -negative patients, with nine other genera trending (*p* ˂ 0.05) towards significance (Table 1; Fig. 1E). DESeq2 analysis identified 25 significantly differentially abundant genera, including lower abundances of *Rothia* and *Prevotella* in COVID-19-positive patients (See Supplementary Table 6, Additional File 1). A machine learning approach (Boruta) was employed for feature selection to identify taxa driving differences between COVID-19-positive and -negative patients. This analysis identified nine genera, including *Anaerococcus*, *Streptococcus*, *Enterococcus*, and *Bacillus*, that were also identified in DESeq2 and ANCOM analyses (See Supplementary Fig. 1, Additional File 2).

**Table 1:** Results of analysis of compositions (ANCOM) between COVID-19-positive and -negative nasopharyngeal samples

ANCOM			
**(Phylum) Genus**	**Median Percentile Abundance: COVID-19-Negative**	**Median Percentile Abundance: COVID-19-Positive**	**W Score**
(Firmicutes) *Streptococcus*	213.92	1.0	**19**
***Genera trending towards significance***			
(Firmicutes) *Enterococcus*	37.25	292	8
(Proteobacteria) *Burkholderia-Caballeronia-Paraburkholderia*	1.0	91.33	8
(Proteobacteria) ***Gulbenkiania***	1.0	33.67	6
(Firmicutes) ***Finegoldia***	121.83	1.0	5
(Firmicutes) ***Anaerococcus***	173.42	1.0	4
(Proteobacteria) ***Neisseriaceae Uncultured***	371.42	1.0	4
(Firmicutes) *Bacillus*	47.58	624.67	4
(Bacteroidetes) ***Prevotella***	70.58	1.0	3
(Actinobacteria) *Corynebacterium*	3,434.08	236.67	3

ANCOM: samples with fewer than 5,000 sequences were removed; features that were not present in at least 4 samples were removed; features with an overall count of less than 500 [10% of feature biom – due to small sample size ≤ 10 per group] were also removed. All listed significant features [genus] rejected the null hypothesis. Median percentile abundance (average of the 25^th^, 50^th^, 75^th^ percentiles) and analysis W scores determined using ANCOM. ANCOM values were corrected for multiple testing using the Benjamini–Hochberg method (*q*-value < 0.05: bold). Taxa also identified as significant in DESeq2 analyses are bolded

## Discussion

This proof-of-concept study provides evidence that current biorepositories with nasopharyngeal VTM can be utilized to assess microbiome communities. Importantly, this study is the first to show that COVID-19-positive patients have a dysbiotic nasopharyngeal microbial community characterized by loss of putative nasal commensal bacteria and an increase in putative pro-inflammatory bacteria. Specifically, this study revealed a characteristic nasal microbiome in COVID-19-positive individuals with (1) a trend toward reduced microbial alpha diversity, (2) an alteration in the relative abundance of multiple taxa within the phylum Proteobacteria and a significantly increased ratio of Proteobacteria:Actinobacteria, and (3) decreased relative abundance of nasal commensal organisms such as *Corynebacterium* (Actinobacteria) [24] and *Streptococcus* (Firmicutes), previously shown to be affected by influenza virus infection [25]. These results need to be further evaluated in future studies using metagenomic and metatranscriptomic techniques, but may reflect baseline differences prior to infection leading to susceptibility to SARS-CoV-2 infection or may be a result of SARS-CoV-2 infection, leading to post-infection microbiome alteration.

Recent cross-sectional studies have attempted to distinguish nasal microbial profiles collected from COVID-19-positive and negative subjects using nasopharyngeal swab [5–8]. Of the four prior studies using nasopharyngeal swabs, our results are most similar to those of *Mostafa* et al. [7]. Mostafa et al., using non-targeted shotgun sequencing approaches, found significant decreases in microbial alpha diversity, significantly different in microbial community structures, and a higher relative abundance of *Propionibacteriaceae* and lower *Corynebacterium accolens* in COVID-19-positive relative to -negative individuals. In comparison, VTM data in this study indicated a similarly reduced (though not significant) microbial diversity, a significant difference between microbial communities, and a markedly decreased abundance of genera *Corynebacterium* in COVID-19-positive relative to -negative patients*.* Due to mismatches between the 16S rRNA gene sequence of bacteria from the genus *Propionibacterium* and the V4 primers employed in this study (*e.g.*, Meisel et al. [26]), *Propionibacterium* were not well represented and no direct comparison with the results of Mostafa et al. could be made. Elsewhere, Nardelli and colleagues targeted the V1-V3 variable regions of 16S rRNA genes using amplicon sequencing [8], while De Maio et al. targeted the V5-V6 variable regions [5]. Differences in PCR conditions can make direct comparison of results generated with different primer sets challenging, as PCR conditions can introduce substantial bias into observed microbial communities. Finally, Braun et al. performed a cross-sectional 16S rRNA sequencing study, with longitudinal sampling from a subset of subjects, and targeting the V4 variable region [6], as performed in this study. However, Braun et al. reported no significant effect of SARS-CoV-2 on the nasopharyngeal microbial community using multiple analysis methods. This proof-of-concept study using VTM revealed significant differences microbiome between COVID-19-positive and COVID-19-negative samples in this study. We note, however, that future analyses will need to also determine the genome sequence of the SARS-CoV-2 virus, as different variants may differentially alter microbial community structure. At the time of sampling for this study, currently circulating variants of concern had not yet arisen.

Limitations include the relatively small sample size that may have constrained our ability to identify additional significant difference between groups. We acknowledge a lack of inclusion of in-depth clinical medical information, as only basic demographic data was collected from patients at the COVID-19 rapid COVID-19 testing sites (i.e., drive-thru or patient drop-off) from where these samples were obtained. Additionally, internal household spousal or live-in close members as COVID-19 negative controls would be an ideal comparison cohort to help exclude environmental effects. We suggest that future research studies should attempt to include these data.

In conclusion, this proof-of concept study demonstrates the feasibility of using VTM from nasopharyngeal swab collections to study nasopharyngeal microbiota in COVID-19. As hospitals have collected and stored COVID-19 testing swabs and VTM over the entirety of the pandemic, this opens a huge potential dataset for interrogating the nasal microbiome. Secondly, this proof-of-concept study provides preliminary data suggesting the presence of a dysbiotic and pro-inflammatory nasopharyngeal microbiota in COVID-19-positive patients. Our study provides a strong scientific rationale for future studies to investigate the relationship between nasal microbiome and SARS-CoV-2 infection and COVID-19 severity, and also the relationship to the long-lasting effects of COVID-19.

## Supplementary Information

**Additional file 1: Supplementary Table 1.** Demographics and clinical characteristics of COVID-19-positive individuals. **Supplementary Table 2.** Alpha-diversity values in nasopharyngeal samples. **Supplementary Table 3.** Global community analysis of nasopharyngeal microbial community structure in COVID-19-positive and -negative patients, as assessed by Permutational Multivariate Analyses of Variance (PERMANOVA). **Supplementary Table4.** Relative abundances of bacterial taxa in COVID-19-positive and -negative nasopharyngeal samples. **Supplementary Table 5.** Differential relative abundance of nasopharyngeal bacterial phyla in COVID-19-positive and -negative samples identified by DESeq2 analysis. **Supplementary Table 6.** Genus taxonomic level differential abundance DeSeq2 analysis between COVID-19-positive and -negative patient’s nasopharyngeal samples.

**Additional file 2: Supplementary Figure1. **Microbial features driving differentiation of COVID-19-positive and -negative patients. A predictive model based on the genus-level relative abundance was generated using Boruta. Green boxes are bacterial genera that are strongly associated with differentiating the groups using the Boruta feature selection algorithm. Blue boxes are the shadow genera introduced into Random Forest classifier to act as benchmarks. Red boxes are bacterial genera that were not associated with differentiating the groups.

## Data Availability

Raw sequence data (FASTQ files) were deposited in the National Center for Biotechnology Information (NCBI) Sequence Read Archive (SRA), under the BioProject identifier PRJNA704967.
