# Supplementary material for: Nasopharyngeal Microbiota in SARS-CoV-2 Positive and Negative Patients
Source: Biol Proced Online. 2021 Jun 1;23:10. doi: 10.1186/s12575-021-00148-6 (PMC8166531; doi:10.1186/s12575-021-00148-6)
Supplement: Supplementary file 1 — Additional file 1: Supplementary Table 1. Demographics and clinical characteristics of COVID-19-positive individuals. Supplementary Table 2. Alpha-diversity values in nasopharyngeal samples. Supplementary Table 3. Global community analysis of nasopharyngeal microbial community structure in COVID-19-positive and -negative patients, as assessed by Permutational Multivariate Analyses of Variance (PERMANOVA). Supplementary Table4. Relative abundances of bacterial taxa in COVID-19-positive and -negative nasopharyngeal samples. Supplementary Table 5. Differential relative abundance of nasopharyngeal bacterial phyla in COVID-19-positive and -negative samples identified by DESeq2 analysis. Supplementary Table 6. Genus taxonomic level differential abundance DeSeq2 analysis between COVID-19-positive and -negative patient’s nasopharyngeal samples. [file 12575_2021_148_MOESM1_ESM.docx]

**Additional Information**

**Supplementary Tables**

**Supplementary Table 1.** Demographics and clinical characteristics of COVID-19-positive individuals.

| **COVID-19 Positive Patient Number** | **SARS-CoV-2 RT-PCR Ct Value** | **Sex** | **Race** | **Ethnicity** | **Age** |
| --- | --- | --- | --- | --- | --- |
| 1 | 27.16 | Male | Other | Hispanic or Latino | 29 |
| 2 | 17.91 | Female | Black or African American | Not Hispanic or Latino | 47 |
| 3 | 28.72 | Female | Black or African American | Not Hispanic or Latino | 42 |
| 4 | 28.64 | Male | Black or African American | Not Hispanic or Latino | 49 |
| 5 | 26.26 | Female | Black or African American | Not Hispanic or Latino | 68 |
| 6 | 8.08 | Male | White | Not Hispanic or Latino | 72 |
| 7 | 15.08 | Female | Other | Hispanic or Latino | 68 |
| 8 | 17.37 | Female | Other | Hispanic or Latino | 52 |
| 9 | 13.03 | - | - | - | - |
| **Summary** | **Mean ± (SD)**  **20.25 ± (7.64)** | **3 - Male**  **5 - Female** | **1 - White;**  **4 - Black or African American;**  **3 - Other** | **3 - Hispanic or Latino;**  **5 - Not Hispanic or Latino** | **Mean ± (SD)**  **53.38 ± (14.93)** |
| COVID-19-positive patients were all collected on the same date in April 2020, via the RUMC drive-thru testing site. No patients were put on ventilation or died. All RT-PCR testing conducted on Abbott *m*2000 device. Ct, cycle threshold; -, unknown information. | | | | | |

**Supplementary Table 2.** Alpha-diversity values in nasopharyngeal samples.

| **Diversity Index** | **COVID-19-Negative**  **Mean** **± (SD)** | **COVID-19-Positive**  **Mean ± (SD)** | **p value** |
| --- | --- | --- | --- |
| Shannon | 3.83 ± (1.11) | 3.58 ± (1.16) | 0.647 |
| Simpson | 0.81 ± (0.12) | 0.76 ± (0.19) | 0.661 |
| Observed Features | 78.80 ± (38.40) | 60.11 ± (23.05) | 0.222 |
| Evenness | 0.62 ± (0.13) | 0.61 ± (0.17) | 0.842 |
| Shannon Index, Simpson’s Index, observed features (richness) and Pielou’s evenness were measured at the feature (ASV) level. Datasets were rarefied to 5,000 sequences per sample. Mean index score and standard deviation (SD) are displayed. COVID-19-negative (n = 10); COVID-19-positive (n = 9). | | | |

**Supplementary Table 3.** Global community analysis of nasopharyngeal microbial community structure in COVID-19-positive and -negative patients, as assessed by Permutational Multivariate Analyses of Variance (PERMANOVA).

| **Comparison** | **Feature Taxonomic Level** | | | |
| --- | --- | --- | --- | --- |
|  | **Sample Size** | **Permutations** | **Pseudo-F** | **q value** |
| COVID-19-Negative vs. COVID-19-Positive | 19 | 9,999 | 2.16 | **0.016** |
| PCR Blank vs. COVID-19-Negative | 15 | 9,999 | 5.30 | **0.002** |
| PCR Blank vs. COVID-19-Positive | 14 | 9,999 | 5.23 | **0.002** |
| PERMANOVA results are based on a Bray-Curtis distance matrix. Significance was determined using 9,999 permutations and corrected for multiple testing using the Benjamini-Hochberg method (q < 0.05). COVID-19-negative (n = 10); COVID-19-positive (n = 9); PCR Reagent Blanks (n = 5). | | | | |

**Supplementary Table 4.** Relative abundances of bacterial taxa in COVID-19-positive and -negative nasopharyngeal samples.

| **Taxonomic Level** | **Negative**  **Mean RA % ± (SD) %** | **Positive**  **Mean RA % ± (SD) %** |
| --- | --- | --- |
| **Phylum** | | |
| Proteobacteria | 38.26 ± (22.27) | 50.19 ± (20.83) |
| Actinobacteria | 27.30 ± (15.44) | 12.56 ± (23.49) |
| Firmicutes | 29.47 ± (21.61) | 35.34 ± (17.90) |
| Bacteroidetes | 3.04 ± (2.35) | 0.72 ± (0.83) |
| Campilobacterota | 1.07 ± (1.60) | 0.07 ± (0.20) |
| **Phylum: *Genus*** | | |
| Actinobacteria: *Corynebacterium* | 19.52 ± (12.36) | 10.64 ± (20.52) |
| Proteobacteria: *Morganella* | 5.19 ± (16.40) | 0.00 ± (0.00) |
| Proteobacteria: *Moraxella* | 5.92 ± (14.30) | 9.32 ± (27.45) |
| Proteobacteria: *Escherichia-Shigella* | 14.08 ± (16.95) | 26.45 ± (15.76) |
| Proteobacteria: *Proteus* | 2.80 ± (8.84) | 0.00 ± (0.00) |
| Firmicutes: *Staphylococcus* | 6.11 ± (5.25) | 12.02 ± (11.58) |
| Firmicutes: *Peptoniphilus* | 3.52 ± (4.50) | 0.64 ± (0.79) |
| Actinobacteria: *Lawsonella* | 4.35 ± (6.88) | 1.21 ± (3.23) |
| Firmicutes: *Bacillus* | 1.02 ± (1.66) | 6.82 ± (5.37) |
| Firmicutes: *Bacillales Unclassified* | 1.76 ± (3.79) | 4.86 ± (3.90) |
| Proteobacteria: *Pseudomonas* | 2.18 ± (1.63) | 4.17 ± (2.49) |
| Firmicutes: *Streptococcus* | 7.03 ± (18.07) | 0.57 ± (1.66) |
| Firmicutes: *Dolosigranulum* | 1.09 ± (2.88) | 0.47 ± (1.34) |
| Firmicutes: *Enterococcus* | 1.21 ± (1.68) | 4.17 ± (3.07) |
| Proteobacteria: *Neisseriaceae Uncultured* | 2.40 ± (2.63) | 0.23 ± (0.69) |
| Firmicutes: *Anaerococcus* | 1.73 ± (1.71) | 0.54 ± (1.28) |
| Campilobacterota: *Campylobacter* | 1.07 ± (1.60) | 0.07 ± (0.20) |
| Bacteroidetes: *Prevotella* | 1.57 ± (1.87) | 0.01 ± (0.02) |
| Firmicutes: *Finegoldia* | 0.85 ± (0.98) | 0.06 ± (0.18) |
| Proteobacteria: *Tepidiphilus* | 0.39 ± (0.77) | 2.02 ± (1.50) |
| Proteobacteria: *Burkholderia-Caballeronia-Paraburkholderia* | 0.39 ± (0.93) | 1.23 ± (0.95) |
| Mean RA % = average number of sequences per taxa, calculated from the total sum of all sequence counts, depicted as a percentage. Microbial taxa (˃ 1%) shown. (SD) % = standard deviation as a percentage. COVID-19-negative (n = 10); COVID-19-positive (n = 9). | | |

**Supplementary Table 5.** Differential relative abundance of nasopharyngeal bacterial phyla in COVID-19-positive and -negative samples identified by DESeq2 analysis.

| **Phylum** | **Base Mean** | **Log2 FC**  **(Positive over Negative)** | **p value** | **q value** |
| --- | --- | --- | --- | --- |
| Bacteroidetes | 319.44 | -2.06 | *0.041* | 0.260 |
| Proteobacteria | 6,640.78 | 0.59 | 0.085 | 0.260 |
| Phyla differential abundances determined using DESeq2. Base Mean = mean of normalized samples. Log2 FC = Log2 fold change of taxa in COVID-19-positive in comparison to -negative samples. DeSeq2 was corrected for multiple testing using the Benjamini-Hochberg method (q-value < 0.05: bold); (p-value < 0.05: italics). COVID-19-negative (n = 10); COVID-19-positive (n = 9). | | | | |

**Supplementary Table 6.** Genus taxonomic level differential abundance DeSeq2 analysis between COVID-19-positive and -negative patient’s nasopharyngeal samples.

| **(Phylum) Genus** | **Base Mean** | **Log2FC**  **(Positive over Negative)** | ***p* or q values** |
| --- | --- | --- | --- |
| (Firmicutes) *Tumebacillus* | 10.33 | 5.79 | *0.0096* |
| (Proteobacteria) ***Gulbenkiania*** | 39.13 | 3.44 | *0.0222* |
| (Firmicutes) ***Anaerococcus*** | 243.47 | -3.24 | *0.0456* |
| (Firmicutes) *Peptoniphilus* | 485.46 | -3.50 | *0.0207* |
| (Proteobacteria) ***Neisseriaceae Uncultured*** | 241.68 | -4.53 | *0.0196* |
| (Bacteroidetes) *Chryseobacterium* | 4.78 | -4.91 | *0.0241* |
| (Proteobacteria) *Selenomonas* | 1.64 | -5.00 | *0.0448* |
| (Proteobacteria) *Moraxella* | 301.70 | -5.02 | *0.0177* |
| (Firmicutes) ***Finegoldia*** | 113.74 | -5.02 | *0.0069* |
| (Bacteroidetes) *Porphyromonas* | 1.77 | -5.08 | *0.0413* |
| (Firmicutes) *Butyricicoccus* | 1.87 | -5.11 | *0.0399* |
| (Campilobacterota) *Campylobacter* | 152.44 | -5.12 | *0.0103* |
| (Firmicutes) *Gemella* | 1.94 | -5.17 | *0.0376* |
| (Bacteroidetes) *Flavobacterium* | 2.05 | -5.22 | *0.0358* |
| (Firmicutes) *Veillonella* | 57.40 | -5.30 | *0.0132* |
| (Proteobacteria) *Vibrio* | 2.24 | -5.33 | *0.0319* |
| (Firmicutes) *Bacilli Unclassified* | 2.43 | -5.41 | *0.0295* |
| (Firmicutes) *Christensenellaceae R-7 group* | 3.60 | -5.82 | *0.0189* |
| (Bacteroidetes) *Capnocytophaga* | 3.54 | -5.85 | *0.0184* |
| (Actinobacteria) *Micrococcus* | 5.28 | -6.38 | *0.0088* |
| (Actinobacteria) *Blastococcus* | 6.68 | -6.67 | *0.0058* |
| (Bacteroidetes) *Alloprevotella* | 6.76 | -6.70 | *0.0054* |
| (Actinobacteria) *Kocuria* | 8.18 | -6.75 | *0.0063* |
| (Bacteroidetes) ***Prevotella*** | 209.16 | -7.82 | **0.0020** |
| (Actinobacteria) *Rothia* | 24.70 | -8.72 | **0.0020** |
| DESeq2: Taxa shown have adjusted p-values (q ˂ 0.05 indicated by bold; p ˂ 0.05 indicated by italics) with a log2 fold change (˃ ± 3) at the level of genus. Base Mean = mean of normalized samples. Log2FC = Log2 fold change of taxa in COVID-19-positive in comparison to -negative samples. Taxa identified by both DESeq2 and ANCOM are bolded above. COVID-19-negative (n = 10); COVID-19-positive (n = 9). | | | |
