# Supplementary figures and images for: Nasopharyngeal Microbiota in SARS-CoV-2 Positive and Negative Patients
Source: Biol Proced Online. 2021 Jun 1;23:10. doi: 10.1186/s12575-021-00148-6 (PMC8166531; doi:10.1186/s12575-021-00148-6)

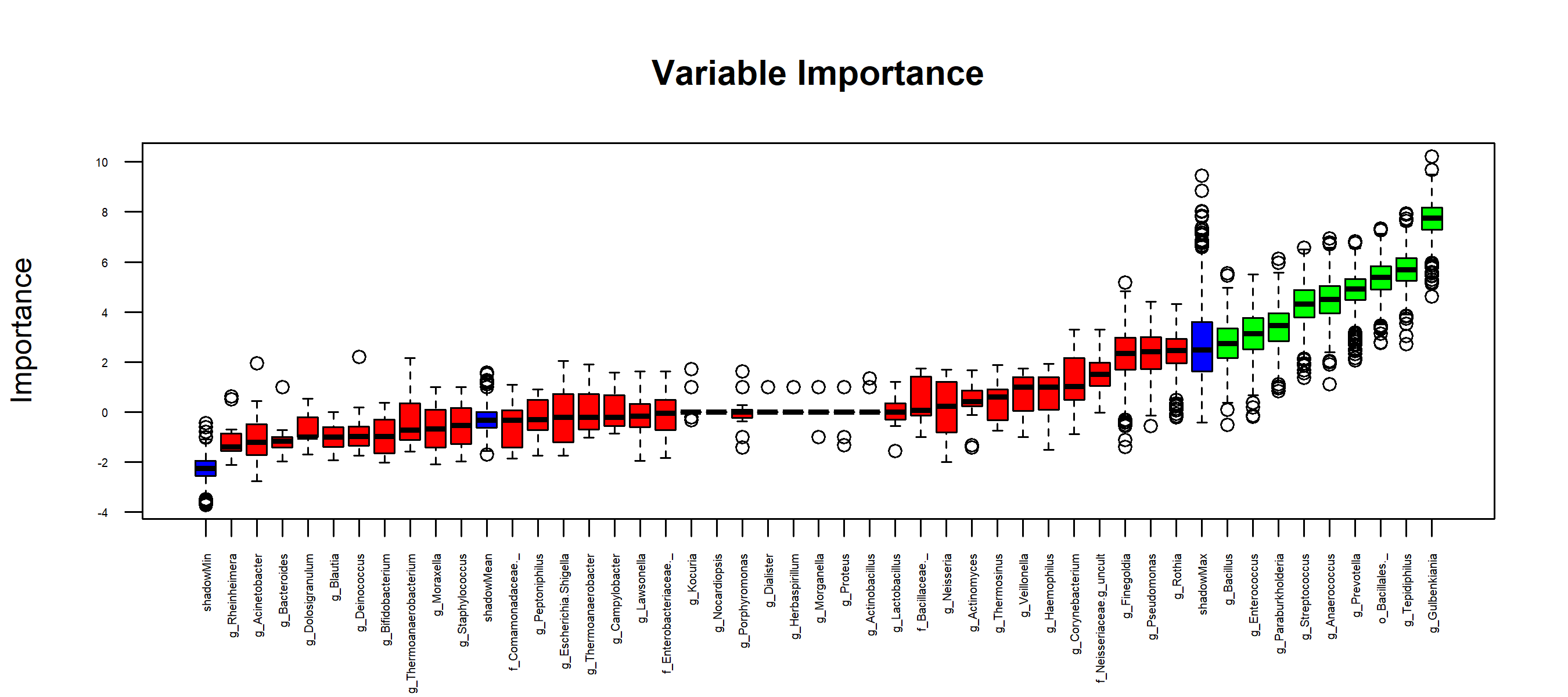

Supplement: Supplementary file 2 — Additional file 2: Supplementary Figure1. Microbial features driving differentiation of COVID-19-positive and -negative patients. A predictive model based on the genus-level relative abundance was generated using Boruta. Green boxes are bacterial genera that are strongly associated with differentiating the groups using the Boruta feature selection algorithm. Blue boxes are the shadow genera introduced into Random Forest classifier to act as benchmarks. Red boxes are bacterial genera that were not associated with differentiating the groups. [file 12575_2021_148_MOESM2_ESM.tiff]
